# Complete Cure of Prolapsus Uteri without Medical Aid

**Published:** 1872-10

**Authors:** 


Complete Cure of Prolapsus Uteri without Mechanical Aid.—Nicolai Andreef, student of medicine at Kasan, in Russia, gives the history of a number of cases of partial and complete prolapsus uteri, in which complete cures were accomplished by the local use of a diluted tincture of iodine. Several of his cases represent extreme forms of that disease, where slight mechanical disturbances—for instance, a paroxysm of cough, walking a few steps, etc.—were sufficient to displace the womb. He paints, after replacing the uterus, the walls of the vagina from the cervix downward, by the aid of the speculum, with tincture of iodine, which has been diluted by the addition of spiritus vini rectificatus. For the first application, he dilutes this tincture with one-half its quantity of spiritus vini; for the following, with one-third of it. At the first application, only one-half drachm, at the following ones about one drachm of the tincture is consumed. The applications are not repeated oftener than every three days, and vaginal douches of cold water are made within half an hour afterward, and repeated four times daily. The horizontal position must be maintained during the intervals. From two to six applications are sufficient to accomplish a cure. The principal points to be observed to make this treatment successful are—

1. The uterus must first be replaced; where that is impossible, this treatment is inadmissible.

2. All lesions of the vagina and uterus—for instance, excoriations, ulcerations, etc.—should first be removed to prevent inflammatory phenomena.

3. Only the walls of the vagina should be painted—first with small quantities and a weak solution, afterward with larger quantities and a stronger solution of the iodine. Cold vaginal douches must be used to mitigate the irritation of the vagina and uterus.

4. After two applications, the strict maintainance of the horizontal position is no longer absolutely necessary.

5. The alimentary canal must be kept clean.

6. The applications must not be made oftener than every three days, and the vaginal douches must be continued some time after their suspension.—*Virchow's Archiv, No.* 4, '72. R.

